# Assessment and Exposure Analysis of Trace Metals in Different Age Groups of the Male Population in Southern Punjab, Pakistan

**DOI:** 10.3390/toxics11120958

**Published:** 2023-11-24

**Authors:** Sajjad Hussain, Tasawar Khanam, Subhan Ullah, Fouzia Aziz, Abdul Sattar, Imran Hussain, Muhammad Abu Bakar Saddique, Amna Maqsood, Changfeng Ding, Xingxiang Wang, Jianjun Yang

**Affiliations:** 1 Institute of Environment and Sustainable Development in Agriculture, Chinese Academy of Agricultural Sciences, Beijing 100081, China; sajjad.husains786@gmail.com (S.H.); subhan_agri@yahoo.com (S.U.); 2 Layyah Institute, University of Lahore, Layyah 31200, Pakistan; 3 Ecohealth and Toxicology Laboratory, Department of Biosciences, COMSATS University Islamabad, Islamabad 44000, Pakistan; tasawwursatti@gmail.com; 4 Department of Zoology, University of Chakwal, Chakwal 48800, Pakistan; 5 Department of Economics, University of Layyah, Layyah 31200, Pakistan; 6 Department of Economics, Women University, Multan 60000, Pakistan; 7 Department of Agronomy, Bahauddin Zakariya University, Multan 60800, Pakistan; abdulsattar04@gmail.com; 8 Environmental Biotechnology Laboratory, Department of Biotechnology, Abbottabad Campus, COMSATS University Islamabad, Abbottabad 22020, Pakistan; imranhussain@cuiatd.edu.pk; 9 Institute of Plant Breeding and Biotechnology, MNS University of Agriculture, Multan 60000, Pakistan; abubakar.siddique@mnsuam.edu.pk; 10 Institute of Soil and Environmental Sciences, PMAS Arid Agriculture University, Rawalpindi 46300, Pakistan; amnamaqsood10@gmail.com; 11 CAS Key Laboratory of Soil Environment and Pollution Remediation, Institute of Soil Science, Chinese Academy of Sciences, Nanjing 210008, China; cfding@issas.ac.cn (C.D.); xxwang@issas.ac.cn (X.W.); 12 Institute of Agricultural Resources and Regional Planning, Chinese Academy of Agricultural Sciences, Beijing 100081, China

**Keywords:** trace metals, arsenic, exposure source, health risks, Pakistan

## Abstract

In developing countries, like Pakistan, the pursuit of urbanization and economic development disrupts the delicate ecosystem, resulting in additional biogeochemical emissions of heavy metals into the human habitat and posing significant health risks. The levels of these trace elements in humans remain unknown in areas at higher risk of pollution in Pakistan. In this investigation, selected trace metals including Copper (Cu), Chromium (Cr), Lead (Pb) Cadmium (Cd), Cobalt (Co), Nickel (Ni), and Arsenic (As) were examined in human hair, urine, and nail samples of different age groups from three major cities (Muzaffargarh, Multan, and Vehari) in Punjab province, Pakistan. The results revealed that the mean concentrations (ppm) of Cr (1.1) and Cu (9.1) in hair was highest in Muzaffargarh. In urine samples, the mean concentrations (μg/L) of Co (93), As (79), Cu (69), Cr (56), Ni (49), Cd (45), and Pb (35) were highest in the Multan region, while As (34) and Cr (26) were highest in Vehari. The mean concentrations (ppm) of Ni (9.2), Cr (5.6), and Pb (2.8), in nail samples were highest in Vehari; however, Multan had the highest Cu (28) concentration (ppm). In urine samples, the concentrations of all the studied metals were within permissible limits except for As (34 µg/L) and Cr (26 µg/L) in Vehari. However, in nail samples, the concentrations of Ni in Multan (8.1 ppm), Muzaffargarh (9 ppm), Vehari (9.2 ppm), and Cd (3.69 ppm) in Muzaffargarh exceeded permissible limits. Overall, the concentrations of metals in urine, nail, and hair samples were higher in adults (39–45 age group). Cr, Cu, and Ni revealed significantly higher concentrations of metals in hair and water in Multan, whereas As in water was significantly (*p* < 0.001) correlated with urinary As in Multan, indicating that the exposure source was region-specific.

## 1. Introduction

Biomonitoring of heavy metals (HMs) in biological samples is crucial for advancing biomedical research and addressing clinical concerns. Biological monitoring provides a means to investigate exposure limits and associated health risks posed by potentially toxic elements. The global attention directed at the elevated concentrations of heavy metals (HMs) in the environment underscores their ubiquitous and toxic nature [1,2,3]. Heavy metals are extremely toxic because of their non-biodegradable nature and potential for bio-accumulation inside the living body [4]. Various tissues, including hair, urine, and nails, have been successfully used as a biomarkers for assessing the environmental load of toxic factors [5,6,7]. Urinary analysis for multi-elemental characterization offers a comprehensive understanding of the total body intake of key elements compared to other biological samples [8]. Samples of nails and hair are considered non-invasive biomarkers, easy to handle, and particularly valuable in depicting long-term exposure to heavy metals [9].

Extensive reports in the literature highlight Pakistan’s fragile geological environment and delicate ecological systems, which are prone to extensive geogenic contamination by heavy metals, ultimately posing risks to health [10,11].

Excessive human activity, such as urbanization and industrialization, further exacerbates the situation, altering local ecologies and impeding economic growth. The Pakistani population faces ubiquitous exposure to various potential heavy metals through various dietary and non-dietary means due to amplified industrialization and urbanization [12]. Human exposure to toxic heavy metals primarily occurs through dust inhalation and the intake of metal-contaminated water, soil, and food. Upon entering the living body, these metals are disseminated to organs such as the liver, brain, kidneys, and bones [13]. The accumulation of heavy metals in body tissues poses severe human health risks, including reproductive failure, genotoxicity, disruption of the nervous system, and digestive problems [14,15]. Certain elements, such as Zn, Cr, Cu, and Ni are vital components of biological systems but can become toxic by altering gene expressions at high concentrations. Others are categorized as toxic factors even in low concentrations [16,17]. Lead (Pb), for instance, is highly toxic even at low concentrations, earning it a top priority designation in the list of hazardous substances due to its toxicity, persistence, and potential for human exposure [18]. Pb is believed to originate from both natural and human activities, causing immune toxicity, anemia, hypertension, renal failure, and permanent damage to the reproductive organ upon exposure. [19,20]. Long-term exposure to cadmium (Cd) may lead to osteoporosis and nephrotoxicity, damaging the hypothalamus-pituitary axis and potentially causing immune and endocrine disorders [21].

Extremely high levels of zinc (Zn) may result in nausea, vomiting, pain, and diarrhea [22]. Copper (Cu), is crucial for normal physiological function, can be toxic when orally ingested in very high doses, causing dizziness, diarrhea, nausea, stomach spasms, kidney and liver impairment, and even death [23]. To date, the content of heavy metals in humans remain unknown in areas at higher risk of environmental pollution in the Punjab Province, Pakistan. Therefore, this study aims to access the heavy metals (Cd, Cr, Co, Ni, Pb, Cu, and As) in biological samples (hair, nail, and urine) of a healthy population to estimate the risks posed to public health through heavy metal exposure via contaminated water and wheat.

## 2. Materials and Methods

### 2.1. Study Areas

Pakistan’s floodplains consist of eroded sediments and soil from high mountainous regions, potentially containing varying amounts of trace metals and nutrients. These elements can significantly impact the chemical composition of ground and surface water systems. The mineralogy and geochemistry of the Indus floodplain (Punjab and Sindh) are profoundly influenced by these mountainous ranges [11]. Various factors, such as land use, geology, topography, and climatic conditions, also contribute to the overall distribution of trace elements and environmental pollutants [24].

The study area includes the province of Punjab, Pakistan’s second largest province, which is known for its fertile plains along the River Indus. Punjab is mostly irrigated and boasts an extensive network of canals. Sampling was conducted in three major cities of Punjab province: Muzaffargarh, Multan, and Vehari. The latitude for Muzaffargarh, Punjab, Pakistan is 30.074377 degrees north and longitude is 71.184654 degrees east. Muzaffargarh covers an area of about 8432 square kilometers (3257 square miles) and is one of the larger cities in the Punjab region, experiencing extreme temperatures ranging from 1 °C to 54 °C with an annual rainfall of approximately 127 mm. Multan, situated at a latitude of approximately 30.1575° degrees north and longitude of 71.5249° degrees east, spans about 133 square kilometers (51.35 square miles). Multan, one of the largest cities in the Punjab region, faces severe climate conditions, with recorded temperatures ranging from −1 °C to 54 °C. Dust storms are frequent in the city. Vehari, located at a latitude of approximately 30.0277° degrees north and longitude of 72.3488° degrees east, covers an area of about 1368 square kilometers (528 square miles) with a population of 7,000,000. Vehari experiences a hot desert climate, characterized by extremely hot and dry summers and relatively mild winters, with recorded temperatures ranging from 1 °C to 50 °C.

### 2.2. Sample Collection

Environmental (water and wheat) and biological (hair, nail, and urine) samples were obtained from rural sites of three districts: Muzaffargarh (*n* = 60), Multan (*n* = 45) and Vehari (*n* = 95). The aim was to assess potential sources of exposure by metals (Cd, Cr, Co, Ni, Pb, Cu, and As). Only males were included in the study due to the challenges of obtaining approval for collecting samples from females and children in rural areas, given social limitations and hesitancy. Moreover, most of the females and children in rural areas are generally hesitant to contribute their samples. All 200 participants, categorized into five age groups (18–24 years, 25–31 years, 32–38 years, and 39–45 years), willingly participated and completed a detailed questionnaire providing information on weight, age, education level, annual family income, profession, daily water intake, rice consumption per week, number of working hours, smoking activity, and residence time (Table 1). Detailed sample collection methods were described previously by Khanam et al. (2020) [25]. Spot urine samples were collected for trace element exposure assessment, while hair, nail, and water samples were collected from each participant. Hair samples (2 g) were collected from the skull using sterilized non-metallic scissors, sealed in aluminum foil, and stored at −20 °C until further analysis. Nail samples were collected in airtight clear plastic bags. For wheat sampling (*n* = 50), the samples were collected from the rural sites where locals had their own agriculture farms (we attempted to source grains directly from local producers, such as farmers’ markets or local farms). During fieldwork, samples were handled with care to ensure quality and prevent cross-contamination. Water samples were collected using pre-cleaned 1 L polyethylene bottles, acidified with HNO_3_ (65%), and stored at −20 °C for subsequent analysis.

### 2.3. Sample Preparation

Before sampling, HNO_3_ 10% (*v*/*v*) (Sinopharm Chemical Reagent Co., Ltd., Beijing, China) was used to acidify all containers, and ultrapure water was used for repeated rinsing. Urine samples were filtered using a 0.22 μm syringe filter, as described by Khanam et al. (2020) [25]. For metal analysis, wheat, hair, and nail samples underwent acid digestion using an established protocol [12]. Approximately 1 g of wheat sample was taken, and 1 mL of HClO_4_ 70% (*v*/*v*) GR grade and 2 mL of HNO_3_ 65% (*v*/*v*) GR grade (Sinopharm Chemical Reagent Co., Ltd., Beijing, China) were added. The following day, 1 mL of H_2_O_2_ 30% (*v*/*v*) GR grade was added to accelerate microwave digestion. Conditions for the rapid microwave digestion system were set at 800 W (10 min) at 120 °C, followed by 800 W (30 min) at 170 °C. For hair and nail samples (1 g ± 0.01 g), 1 mL of H_2_O_2_ 30% (*v*/*v*) GR grade and 1 mL of HNO_3_ 65% (*v*/*v*) GR grade were added. After digestion, each sample was filtered through a nylon syringe filter (0.22 μm), and the volume was adjusted to 10 mL using milli-Q water for further analysis [12]. Water samples were filtered by using a 0.45 mm filter (cellulose acetate) and trace element analysis was performed by ICP-MS (Inductively Coupled Plasma Mass Spectrometry) as described by [11].

### 2.4. Trace Element Analysis

Potential trace metals (Cd, Cr, As, Co, Ni, Pb, and Cu) were analyzed using inductively coupled plasma mass spectrometry (ICP-MS, Agilent Technologies, Santa Clara, CA, USA). The instrumental parameters were as follows: carrier gas 1.1 L min^−1^, RF power 1510 W, helium gas 3.5 mL min^−1^, nebulizer pump 0.1 RPS, and makeup gas 0.10 L min^−1^. A standard stock solution with a known concentration (100 μg/mL^−1^) of all targeted trace metals was provided by the NCATN (National Center of Analysis and Testing for Nonferrous Metals and Electronic Materials), China.

### 2.5. Quality Control and Assurance

To assess instrument stability, a quality control (QC) sample was used after every set off ten (10) samples. This QC sample comprised aliquots from each sample, providing a representative mix of the entire sample set. Metal levels in all QCs showed a <10% variation. Additionally, spiked samples were obtained by spiking (Cd, Cr, As, Co, Ni, Pb, and Cu) before digestion at two final levels (10, 20 ng mL^−1^) to measure recovery ratios. Everyday working solutions were prepared through suitable dilutions of the standard stock solution using HNO_3_ 65% (*v*/*v*), H_2_O (*v*/*v*/*v* = 1:1:3), and H_2_O_2_ 30% (*v*/*v*) mixtures. The relative difference percentage was <5% for replicated analyses. A randomized style was adopted to run each sample, minimizing uncertainty from injection order artifacts and instrument sensitivity changes throughout the process.

### 2.6. Statistical Analysis

Descriptive analysis was conducted using the Statistical Package for Social Sciences (SPSS version 24), with data visualization through Microsoft Office Excel 2016. One-way analysis of variance (ANOVA) was employed to test differences in heavy metal contents in hair, nail, and urine samples among age groups and between water and wheat samples. Regression analysis was performed for metal concentrations in water, hair, nail, and urinary samples to gain a better understanding of environmental contributions.

## 3. Results

This study involved the examination of 200 male individuals. The details are shown in (Table 1).

The average age and weight of the participants was 28 years (range: 18–45), and 62 kg, respectively. Approximately 10% of the subjects belonged to a high socioeconomic status, while about 60% were engaged in agricultural and labor activities with a low-income status. Among the participants, 140 (70%) were smokers, and 60 (30%) were non-smokers.

**Figure 1:**
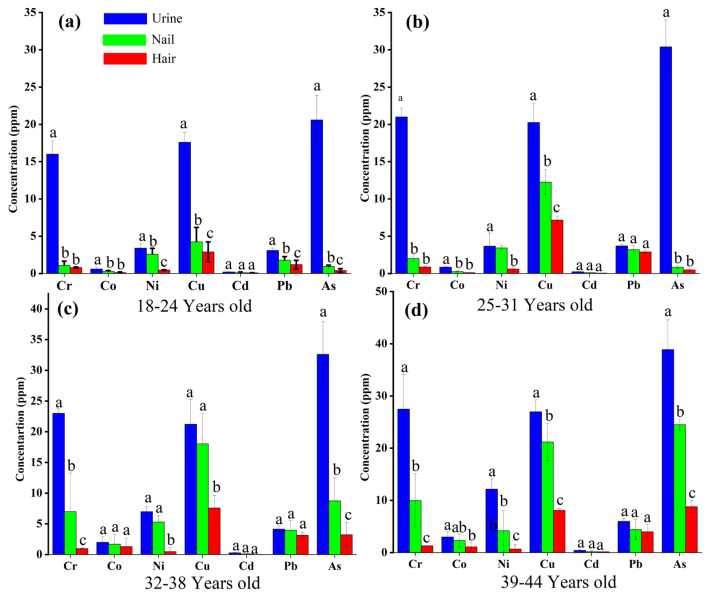
Comparison of hair, urine, and nail metal concentrations between different age groups: 18–24 years (**a**) 25–31 years (**b**), 32–38 years (**c**) and 39–45 years (**d**). Different letters (a–c) on the top of each bar show significant differences among different heavy metals concentration.

A comprehensive overview of the main outcomes, categorized by age groups, is presented in Figure 1. The mean concentration of arsenic (As) in urine samples was highest in the 39–45 age group, exhibiting a gradual increase from 28.6 μg/L in the 18–24 age group to 38.9 μg/L in the 39–45 age group. Additionally, cadmium (Cd) showed its lowest concentration (0.2 μg/L) in the 18–24 age group. In nail samples, the mean concentration (ppm) of copper (Cu) increased gradually from 4.3 in the 18–24 age group to 21 in the 39–45 age group, while As dominated with the highest concentration (25 ppm) in the 39–45 age group. In hair samples, Cu was the dominant metal, with concentrations (ppm) increasing gradually from 2.9 in the 18–24 age group to 8.1 in the 39–45 age group. Additionally, the As concentration (8.8 ppm) was highest in the 39–45 age group’s hair samples. Overall, heavy metal concentrations in hair, urine, and nail samples were higher in the 39–45 age group.

The results of our study revealed higher concentrations of the investigated elements in hair samples from Vehari, Multan, and Muzaffargarh, ranked as Cu > Pb > Cr > Ni > As > Co > Cd. The average concentrations of the studied elements in urine samples followed the order: As > Cr > Cu > Ni > Pb > Co > Cd, while in nail samples, the order was: Cu > Ni > Cr > Pb > As > Co > Cd (Table 2).

**Figure 2:**
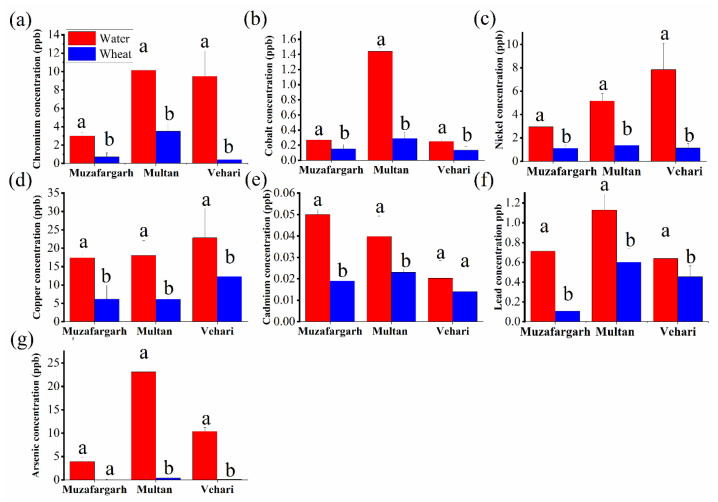
Concentration of (**a**) Cr, (**b**) Co, (**c**) Ni, (**d**) Cu, (**e**) Cd, (**f**) Pb, and (**g**) As in water and wheat in the studied areas. Different letters (a, b) on the top of each bar show significant differences between different heavy metal concentrations.

Hair samples showed higher conc. (ppm) of Cu (9.1) and Cr (1.1) in Muzaffargarh; Co (0.6), Ni (0.49), and As (0.6) in Vehari; and Cd (0.08) and Pb (3.47) in Multan. Urine samples showed elevated conc. (μg/L) of Co (93), As (79), and Cu (69), in Multan while Vehari had higher Cr (26) and As (34). Nail samples recorded elevated conc. (ppm) of Ni (9.2), Cr (5.6), Pb (2.8), and As (2.0) in Vehari while Multan and Muzaffargarh had higher Cu (28) and Ni (9), respectively (Table 3). In the environmental samples, Multan water had higher conc. (μg/L) of Cr (10), Co (1.44), As (23), Pb (1.12), and Cd (0.03), while Vehari showed higher Cu (18) and Ni (8.0). Multan wheat samples had higher conc. (ppm) of Cu (12), Cr (3.5), Pb (0.6), Co (0.29), As (0.08), Ni (1.37), and Cd (0.029) (Figure 2).

Overall, this investigation revealed that the concentration of heavy metals in water and wheat samples was higher in Multan, followed by Vehari and Muzaffargarh. To understand the trend of exposure to wheat and water, regression analysis was conducted. The results showed a highly significant relationship between metals in hair and water samples, specifically Cr, Ni, and Cu in Multan, while water As (*p* < 0.001) was significantly correlated with urinary As in Multan, suggesting that the exposure source was region-specific (Table 4). Moreover, a highly significant correlation between As in drinking water supplies and urine samples in Multan indicated that drinking water was the main source of exposure to As (Table 4).

However, no significant association was observed between wheat, hair, nail, and urine samples. In summary, the levels of heavy metals in the hair, urine, and nail samples were higher in Multan, followed by Vehari and Muzaffargarh. A significant correlation of heavy metals in urine, hair, and water samples was consistently observed in the Multan region.

## 4. Discussion

In this study, we investigated, selected trace metals including Copper (Cu), Chromium (Cr), Lead (Pb) Cadmium (Cd), Cobalt (Co), Nickel (Ni), and Arsenic (As) in human hair, urine, and nail samples across different age groups in three major cities (Muzaffargarh, Multan, and Vehari) of the Punjab province, Pakistan. Our findings revealed a wide range of metals concentrations, with the highest levels observed in Multan, followed by Vehari and Muzaffargarh. Notably, a highly significant association of trace metal contents in hair, urine, and water samples was observed in the Multan region.

Humans experience trace metal exposure in various ways, as effluents emitted in various forms from diverse sources into the local environment (air, water, soil, and food) may pose potential health risks to the inland population. As highlighted by Hussain et al. (2022) and Zafar et al. (2015), higher Pb and As pollution in the soil have significant implications for health safety in Pakistan. Further, the overarching threats of increasing population, climate change, and environmental pollution compound the challenges to food security [28,29,30].

In our investigation, Cu concentration in hair samples was highest in Muzaffargarh, followed by Pb, Cr, Ni, As, Co, and Cd. Copper (Cu) derives from natural sources such as volcanic eruptions, forest fires, sea sprays, and vegetation decay [23,31], while anthropogenic sources include domestic waste water, fossil fuel combustion, waste dumps, and phosphate fertilizers [23].

A significant association between hair and water Cu concentrations was noted in the Multan region, where participants were situated near Cu emission sources like smelters and refineries contributing to elevated exposure. Copper, as a component of an antioxidant enzyme protecting the body from free radical creation, may cause oxidative stress if imbalanced in the body’s metabolism. Excess Cu is absorbed, bound to metallothionein, deposited in the liver, and interferes with cell production through free radical damage [32]. Overall, concentrations of the studied metals in hair samples were within the permissible limits by NHANES (2011) [26] and WHO (1996) [27], except for Ni. Ni concentrations in hair samples from Multan, Muzaffargarh, and Vehari exceeded permissible limits, with a significant association between Ni content in hair, nail, and water samples in the Multan and Vehari regions, suggesting drinking water as a prominent source of Ni exposure. Our Ni assessment results were analogous to or lesser than those observed universally (Table 5). The higher concentration of Ni in hair could be attributed to increased consumption of fatty foods, as hydrogenated oil is a rich Ni source [33]. According to the literature, Ni is a key component of emissions from various industries, and tobacco use, prevalent in 70% of the studied individuals, is a harmful source of Ni due to nickel carbonyl found in cigarettes (Table 1) [34,35].

Although Ni is a natural component of the human diet, it may cause various health issues at higher concentrations (i.e., asthma, birth defects, lung fibrosis, respiratory difficulties, vomiting, and respiratory cancer) [75]. Aside from anthropogenic and natural pollution sources, variations in human metabolism result in different metal accumulations in the body (in urine, hair, and nails). Additionally, Zahm et al. (1992) [76] found that using hair tonics, cosmetics, and hair colors, containing considerable amounts of trace elements, can contribute to high hair metal levels. In nail samples, the concentrations of Ni in Multan, Muzaffargarh, and Vehari, and Cd in Muzaffargarh were found to be elevated than NHANES (2011) [26] and WHO (1996) [27] permissible limits.

The concentrations of Cd and Ni in our study were higher than levels reported in Russia [36], Thailand [37], Nigeria [7], India [43], Sweden [44], but lower than those in Malaysia [40] (Table 5). It has been reported that a single cigarette contains about 1–2 µg of Cd that could be inhaled as 70% of the participants were smokers (Table 1). Atmospheric deposition, natural weathering processes, batteries, phosphate fertilizer usage, municipal solid waste incineration, and sewage treatment plants, among other sources, release Cd into the environment [77,78]. Increased Cd content indicate widespread usage of Cd in batteries, paints, plating industries, and fertilizers [78]. Notably, agricultural soil in Pakistan has recently been identified as Cd-contaminated [30], which causes release of the metal into the atmosphere [79]. Cd causes peroxidation of lipids, catalysis of reactive oxygen species, the generation of inflammatory cytokines, glutathione depletion, and the formation of nitric oxide [80].

These results highlight the health risks posed by trace metal ingestion for inland inhabitants, consistent with conclusions drawn from urine, nail, and hair analysis results. Water is considered the major route by which trace metals produce hazardous health effects, emphasizing the need for efforts to reduce trace metal contents in drinking water in Punjab, Pakistan, through proper purification systems and trace metal load control. Furthermore, wastewater treatment plants must be appropriately utilized to protect the inland population and reduce human health risks. In urine samples, the concentration of all the studied metals was within the NHANES (2011) [26] and WHO (1996) [27] permissible limits except for As and Cr in Vehari. Urinary As concentrations were slightly within the range found in India (38 µg/L) [81]. Arsenic can cross the blood–brain barrier and directly affect the central nervous system [82].

Moreover, studies in the US and Asia have reported adverse health effects in areas with high drinking-water As levels (>1000 µg/L), including cancers, skin lesions, developmental neurological, endocrine, and heart diseases [83,84].

A significant association between urinary and water As levels was observed in the Multan region, suggesting drinking water is a prominent source of As exposure. The observed correlation between As in drinking water and urinary As suggests that the contaminated water is a likely source of As exposure for the affected individuals. This correlation is consistent with what is expected in areas with As-contaminated water sources. In Pakistan, where most people consume untreated groundwater, As exposure is heightened, necessitating focused efforts to reduce metal and metalloid exposure from water, food, and dust. These findings contribute significantly to understanding ongoing pollutant exposure among Pakistani males, emphasizing the need for future public health and research endeavors to address metal and metalloid exposure comprehensively.

## 5. Conclusions

The current investigation underscores the significance of human hair, urine, and nail samples as effective markers for trace metal pollution in the environment. Our findings indicate that the concentrations of trace metals in urine, hair, and nail samples were highest in Multan, followed by Vehari and Muzaffargarh. Notably, a highly significant association between trace metal content in hair, urine, and water samples was observed in the Multan region. The mean metal concentrations observed in our study were comparable to, and in some cases slightly higher than, those reported in the global literature. Across all three sample types, the concentration of metals exhibited a discernible increase in the adult age group (39–45). Arsenic (As), Copper (Cu), and Nickel (Ni) emerged as the primary metal pollutants in the water, serving as potential exposure mediums for metal contamination in the human body. Our findings contribute valuable baseline data on metal toxicity across major districts in Pakistan, showcasing the effectiveness of utilizing human hair, urine, and nail samples as a reliable early warning system for trace metal pollution.

While our study sheds light on the current circumstances, further research is imperative to elucidate the sources of trace metals in both human and natural environments. Investigating the origins of these trace metals will enhance our understanding of the pathways through which they enter the ecosystem and impact human health.

## Figures and Tables

**Table 1:** Sociodemographic characteristics of the study population. expressed as min–max ^a,b^ expressed as n (%).

Factors	Study Population
No of participants	200
Age (years)	28 (18–45) ^a^
Weight	62 (25–110) ^a^
Non-educated	80 (40%) ^b^
Educated	120 (60%)
Smoker	140 (70%) ^b^
Non-smoker	60 (30%) ^b^
High-income	20 (10%) ^b^
Moderate-income	60 (30%) ^b^
Low-income	120 (60%) ^b^
Agricultural worker/Laborer	120 (60%) ^b^
Industrial worker	46 (23%) ^b^
Educational sector	20 (10%) ^b^
No work	14 (7%) ^b^

**Table 2:** Descriptive statistics of analyzed metals in the studied population (*n* = 200).

Sample Type	Metal	Interquartile Range	Mean ± SD	Median	Range	NHANES/WHO *
Hair	Cr	0.4–1.1	0.85 ± 0.67	0.71	5.44	1.2
	Co	0.04–0.1	0.35 ± 3.4	0.07	46.6	0.3
	Ni	0.26–0.5	0.48 ± 0.34	0.4	2.8	0.2
	Cu	5.7–7.8	7.5 ± 8.8	6.8	123	25
	Cd	0.01–0.04	0.04 ± 0.07	0.02	0.62	1
	Pb	0.68–2.9	2.68 ± 4.08	1.48	40	20
	As	0.19–0.5	0.45 ± 0.57	0.32	6.38	-
Urine	Cr	19–26	26 ± 21	23	244	0.22
	Co	0.38–0.9	0.9 ± 2	0.6	25	0.35
	Ni	2.07–4.7	3.89 ± 4	3.1	48	3
	Cu	10.4–27	21 ± 18.2	17	17	-
	Cd	0.13–0.35	0.3 ± 0.5	0.2	5	0.2
	Pb	2.25–4	3.6 ± 3	3	18	0.5
	As	14–46	34 ± 31	25	171	8.6
Nail	Cr	1–2.45	3.14 ± 5.2	1.8	22.4	
	Co	0.1–0.6	0.66 ± 1.27	0.4	5.5	0.3
	Ni	1.9–7.05	8.6 ± 13.4	3.6	47.8	0.2
	Cu	5.05–11.7	14.1 ± 24.5	5.8	105	25
	Cd	0–0.1	0.08 ± 0.19	0	0.8	1.9
	Pb	0.8–4.5	3.1 ± 3.8	1.5	13	20
	As	0.01–1.45	1.1 ± 1.13	1	4.4	-

* Concentrations in ppm for hair and nails, µg/L for urine and similar units for reference values by NHANES, 2011 [26] and WHO, 1996 [27].

**Table 3:** Descriptive statistics of the analyzed metals in hair (ppm), urine (µg/L), and nail (ppm) samples.

		Hair			Urine			Nail		
Metals	Descriptive Stats	Multan	Muzaffargarh	Vehari	Multan	Muzaffargarh	Vehari	Multan	Muzaffargarh	Vehari
Cr	Median	0.4	0.9	0.6	23	24	20	3	1.7	1.9
	Max	3	6	3	284	146	249	3	6	22
	Mean ± SD	0.56 ± 0.5	1.1 ± 0.8	0.8 ± 0.6	56 ± 527	26 ± 16.27	26.92 ± 25	1.78 ± 0.8	2.31 ± 2.46	5.6 ± 9.37
Co	Median	0.1	0.05	0.1	0.54	0.90	0.48	1	0.3	0.3
	Max	1	1	47	662	5	25	1	1	6
	Mean ± SD	0.09 ± 0.1	0.09 ± 0.07	0.6 ± 5	93 ± 118	1.15 ± 1.01	3.19 ± 2.6	0.42 ± 0.2	0.39 ± 0.39	1.3 ± 2.3
Ni	Median	0.3	0.3	0.4	3.61	3.63	2.63	7	2.7	2.7
	Max	2	2	3	1832	48	14	20	48	36
	Mean ± SD	0.5 ± 0.37	0.45 ± 0.3	0.49 ± 0.36	49 ± 371	15. ± 6.2	17.35 ± 1.9	8.1 ± 7	9 ± 17	9.2 ± 15
Cu	Median	6.8	7.4	6.5	16	21.17	13.10	9.3	5.8	5.8
	Max	11	123	25	1068	168	85	105	20	33
	Mean ± SD	6.89 ± 1.79	9.1 ± 15	6.83 ± 2.8	69 ± 179	28 ± 24	34.26 ± 13	28 ± 44	6.18 ± 6.8	12 ± 12
Cd	Median	0	0.015	0	38.4	0.22	0.18	0.1	0.01	0.1
	Max	1	1	1	1144	146	1	1	13	1
	Mean ± SD	0.08 ± 0.12	0.03 ± 0.03	0.05 ± 0.0	45 ± 242	26 ± 26.1	0.22 ± 0.1	0.16 ± 0.3	3.69 ± 4.6	0.0 ± 0.08
Pb	Median	2.0	1.446	1.4	0.50	3.29	2.64	0.01	2.0	1.2
	Max	18	40	12	1837	5	13	2	3	1.2
	Mean ± SD	3.47 ± 4.17	2.99–5.7	2.25 ± 2.5	35 ± 41	0.36 ± 0.7	3.12 ± 1.7	1.5 ± 4	0.81 ± 0.92	2.8 ± 3.24
As	Median	0.4	0.17	0.4	3.01	17	32	0.5	1.0	1.5
	Max	1	1	6	624	19	123	1	2	4
	Mean ± SD	0.45 ± 0.24	0.23 ± 0.16	0.6 ± 0.74	79 ± 127	4.15 ± 3.09	34.26 ± 20	0.7 ± 0.67	0.03 ± 0.04	2 ± 1.4

**Table 4:** Standardized regression coefficient reflecting the relationship between hair, nail, and urinary metals concentrations and their levels in water and wheat.

		Multan		Muzaffargarh		Vehari	
Dependent Variable	Independent Variable	Β	*p*-Value	Β	*p*-Value	β	*p*-Value
Hair Cr	Cr_water_	28	<0.001	−17	0.63	0.26	0.9
	Cr_wheat_	−0.2	0.3	−0.69	0.1	−0.69	0.29
Hair Co	Co_water_	−6.6	0.93	19	0.76	8	0.83
	Co_wheat_	0.57	0.113	0.65	0.02	−1.77	0.02
Hair Ni	Ni_water_	43	0.01	−24	0.28	−0.23	0.89
	Ni_wheat_	0.5	0.13	2.04	0.1	0.76	0.03
Hair Cu	Cu_water_	175	0.01	−11	0.93	9	0.41
	Cu_wheat_	0.30	0.074	1.03	0.05	−1.36	0.6
Hair Cd	Cd_water_	868	0.45	−64	0.78	69	0.82
	Cd_wheat_	0.30	0.001	−0.21	0.19	−2.9	0.1
Hair Pb	Pb_water_	251	0.15	−20	0.93	416	0.51
	Pb_wheat_	−1.09	0.31	0.75	0.21	1.33	0.36
Hair As	As_water_	−0.51	0.89	−0.72	0.81	−5.73	0.54
	As_wheat_	0.57	0.11	0.65	0.01	−1.77	0.02
Urinary Cr	Cr_water_	−0.20	0.38	−0.48	0.50	−0.20	0.38
	Cr_wheat_	−5.92	0.01	3.41	0.06	−0.45	0.23
Urinary Co	Co_water_	−0.28	0.20	−0.33	0.71	−0.28	0.20
	Co_wheat_	−0.09	0.52	−1.77	0.02	0.31	0.03
Urinary Ni	Ni_water_	−0.10	0.26	−0.62	0.16	−0.10	0.26
	Ni_wheat_	0.9	0.002	0.32	0.02	−11.91	0.33
Urinary Cu	Cu_water_	1.38	0.50	0.76	0.19	−0.60	0.40
	Cu_wheat_	0.76	0.19	−0.2	0.8	1.38	0.5
Urinary As	As_water_	4.5	<0.001	−0.39	0.64	−0.39	0.64
	As_wheat_	0.65	0.019	−1.77	0.02	0.57	0.11
Urinary Cd	Cd_water_	4.38	0.58	3.4	0.4	4.38	0.58
	Cd_wheat_	−11.9	0.331	−0.60	0.40	−0.17	0.43
Urinary Pb	Pb_water_	−0.82	0.59	0.6	0.21	−0.82	0.59
	Pb_wheat_	4.96	0.013	1.29	0.23	−0.92	0.62
Nail Co	Co_water_	−0.34	0.12	0.26	0.8	−0.06	0.54
	Co_wheat_	1.38	0.5	0.76	0.19	−0.2	0.8
Nail Cr	Cr_water_	9.1	0.49	−0.16	0.6	−0.06	0.43
	Cr_wheat_	−0.09	0.52	−1.98	0.015	−1.77	0.02
Nail Ni	Ni_water_	0.32	0.27	−0.05	0.25	0.075	0.03
	Ni_wheat_	2.04	0.1	−0.51	0.46	−0.38	0.801
Nail Cu	Cu_water_	0.1	0.53	0.11	0.11	−0.21	0.5
	Cu_wheat_	1.38	0.5	0.76	0.19	−0.2	0.8
Nail As	As_water_	−0.12	0.72	0.13	0.13	−0.12	0.9
	As_wheat_	0.19	0.41	0.2	0.57	0.005	0.99
Nail Cd	Cd_water_	−0.09	0.01	−0.31	−0.31	0.04	0.3
	Cd_wheat_	-	-	−5.92	0.09	−0.45	0.23
Nail Pb	Pb_water_	−0.00	0.1	0.001	0.001	−0.07	0.58
	Pb_wheat_	−0.39	0.86	0.04	0.91	0.99	0.18

**Table 5:** Comparison of nail (ppm), urine (µg/L), and hair (ppm) metal levels in different countries.

Nails	Country	Cd	Cr	Cu	Ni	Pb	Zn	Reference
	Russia	0.15	<10	2.9	<2	0.29	259	Savinov et al., 2020 [36]
	Thailand	0.02	-	-	-	9.574	-	Wongsasuluk et al., 2018 [37]
	Iran	-	-	6.5	-	-	158	Janbabai et al., 2018 [38]
	Pakistan	0.14	-	20.72	10.68	10.57	251	Mohmand et al., 2015 [12]
	Vietnam	0.28	-	-	-	1.57	-	Sanders et al., 2014 [39]
	Malaysia	34.57	-	-	95.21	66.74	-	Saat et al., 2013 [40]
	Nigeria	4.54	-	4	16.37	55.67	70	Abdulrahman et al., 2012 [7]
	Kenya	0.73	-	-	-	27.5	95	Were et al., 2008 [41]
	Egypt	0.89	-	6.06	-	12.86	1.55	Rashed and Hossam, 2007 [42]
	India	1.42	87.9	11.62	32.26	53.67	212	Mehra and Juneja, 2005 [43]
	India	0.99	86.62	7.63	56.24	20.21	180	Mehra and Juneja, 2005 [43]
	Sweden	-	-	4.90	-	-	79	Gerhardsson et al., 2002 [44]
	Sweden	0.06	0.76	7.6	0.84	1.06	116	Rodushkin and Axelsson, 2000 [45]
Urine	USA	-	1.78	7.88	-	-	356	Ingle et al., 2017 [46]
	Nigeria	0.005	0.02	-	0.082	0.15	-	Sani and Abdullah, 2017 [47]
	China	0.001	-	0.81	-	0.03	0.81	Tang et al., 2016 [48]
	UK	0.13	0.35	8.75	1.99	0.47	80	Morton et al., 2014 [49]
	Nigeria	0.068	-	-	-	0.24	-	Lawal, 2014 [50]
	Poland	15	35.4	118	44.1	24	556	Brodzka et al., 2013 [51]
	Belgium	0.28	0.13	8.18	2.05	0.87	256	Hoet et al., 2013 [52]
	Swaziland	-	-	-	-	0.040	-	Okonkwo et al., 2001 [53]
	Pakistan	0.37	17.22	-	4.70	3.82	-	Khanam et al., 2020 [25]
	Reference value	0.23	0.10–0.22	-	1.3	0.49	-	NHANES, 2011 [26]
Hair	Thailand	0.07	-	-	-	3.86	-	Wongsasuluk et al., 2018 [37]
	Iran	-	-	13.3	-	-	256	Janbabai et al., 2018 [38]
	Pakistan	0.13	1.02	11.64	7.74	8.08	255	Mohmand et al., 2015 [12]
	Malaysia	23.21	-	-	36.21	37.59	-	Saat et al., 2013 [40]
	Poland	0.11	37	12.35	0.84	1.05	156	Chojnacka et al., 2012 [54]
	Italy	0.16	0.48	59.7	1.75	3.03	329	Dongarra et al., 2011 [55]
	Brazil	0.013	-	-	-	0.34	-	Carneiro et al., 2011 [56]
	Pakistan	1.67	2.34	21.08	4.3	15.50	140	Pasha et al., 2010 [57]
	China	0.55	1.32	40	1.52	49.5	-	Wang et al., 2009 [58]
	Syria	-	-	15.6	2.58	10.7	260	Khuder et al., 2008 [59]
	Egypt	0.53	-	8.76	-	7.32	179	Rashed and Hossam, 2007 [42]
	Korea	0.2	0.9	2.5	-	3	130	Park et al., 2007 [60]
	France	0.01	0.20	20.3	0.23	0.41	162	Goulle et al., 2005 [61]
	India	0.32	-	-	-	7.60	182.4	Mehra and Juneja, 2005 [43]
	Nigeria	1.0	35.1	117.2	26.4	63.6	146.2	Nnorom et al., 2005 [62]
	Spain	0.89	0.88	27.19	-	-	0.41	Pereira et al., 2004 [63]
	Turkey	0.67	-	-	-	3.06	-	Sasmaz et al., 2003 [64]
	India	2.09	35	9.7	6.48	24.8	265	Vishwanathan et al., 2002 [65]
	India	0.61	-	22.54	1.60	4.1	123	Rao et al., 2002 [66]
	Turkey	-	-	60.22	-	-	176	Ulvi et al., 2002 [67]
	Sweden	-	-	16.60	-	-	233	Gerhardsson et al., 2002 [44]
	Egypt	0.82	-	-	-	9.7	-	Mortada et al., 2002 [68]
	Hong Kong	-	-	14.29	-	7.4	210	Man and Zheng, 2002 [69]
	Sweden	0.35	33	293	28	7.26	198	Rodushkin and Axelsson, 2000 [45]
	Poland	0.56	0.4	7.2	0.6	4.8	132	Nowak and Chmielnicka, 2000 [70]
	Hong Kong	-	-	20.14	-	12.04	19	Man et al., 1996 [71]
	Italy	-	-	21	32	8.7	314	Sturaro et al., 1993 [72]
	South America	12.61	1.88	-	-	-	-	Nagra et al., 1992 [73]
	Sudan	-	-	22.1	-	17	170	Eltayeb and Van-Grieken, 1989 [74]
	Pakistan	0.13	1.02	11.64	8.08	8.08	-	Mohmand et al., 2015 [12]
	Reference value	0.25–1.0	0.3–1.2	15–25	0.02–0.2	2–20	-	WHO, 1996 [27]

## Data Availability

The original data presented in the study are included in the article; further inquiries can be directed to the corresponding author.
